# Application of logistic differential equation models for early warning of infectious diseases in Jilin Province

**DOI:** 10.1186/s12889-022-14407-y

**Published:** 2022-11-04

**Authors:** Tianlong Yang, Yao Wang, Laishun Yao, Xiaohao Guo, Mikah Ngwanguong Hannah, Chan Liu, Jia Rui, Zeyu Zhao, Jiefeng Huang, Weikang Liu, Bin Deng, Li Luo, Zhuoyang Li, Peihua Li, Yuanzhao Zhu, Xingchun Liu, Jingwen Xu, Meng Yang, Qinglong Zhao, Yanhua Su, Tianmu Chen

**Affiliations:** 1 State Key Laboratory of Molecular Vaccinology and Molecular Diagnostics, School of Public Health, Xiamen University, 4221-117 South Xiang’an Road, Xiang’an District, Xiamen, Fujian Province People’s Republic of China; 2 Jilin Provincial Centre for Disease Control and Prevention, ChangchunJilin, China, 3145 Jing Yang Road, Green Park District, Changchun, Jilin Province People’s Republic of China; 3 Yaounde Central hospital, Yaounde, Cameroon

**Keywords:** Logistic differential equation model, Generalized logistic differential equation model, Mathematical model, Infectious diseases, Early warning, Jilin province

## Abstract

**Background:**

There is still a relatively serious disease burden of infectious diseases and the warning time for different infectious diseases before implementation of interventions is important. The logistic differential equation models can be used for predicting early warning of infectious diseases. The aim of this study is to compare the disease fitting effects of the logistic differential equation (LDE) model and the generalized logistic differential equation (GLDE) model for the first time using data on multiple infectious diseases in Jilin Province and to calculate the early warning signals for different types of infectious diseases using these two models in Jilin Province to solve the disease early warning schedule for Jilin Province throughout the year.

**Methods:**

Collecting the incidence of 22 infectious diseases in Jilin Province, China. The LDE and GLDE models were used to calculate the recommended warning week (RWW), the epidemic acceleration week (EAW) and warning removed week (WRW) for acute infectious diseases with seasonality, respectively.

**Results:**

Five diseases were selected for analysis based on screening principles: hemorrhagic fever with renal syndrome (HFRS), shigellosis, mumps, Hand, foot and mouth disease (HFMD), and scarlet fever. The GLDE model fitted the above diseases better (0.80 ≤ *R*^*2*^ ≤ 0.94, *P* <  0. 005) than the LDE model. The estimated warning durations (per year) of the LDE model for the above diseases were: weeks 12–23 and 40–50; weeks 20–36; weeks 15–24 and 43–52; weeks 26–34; and weeks 16–25 and 41–50. While the durations of early warning (per year) estimated by the GLDE model were: weeks 7–24 and 36–51; weeks 13–37; weeks 11–26 and 39–54; weeks 23–35; and weeks 12–26 and 40–50.

**Conclusions:**

Compared to the LDE model, the GLDE model provides a better fit to the actual disease incidence data. The RWW appeared to be earlier when estimated with the GLDE model than the LDE model. In addition, the WRW estimated with the GLDE model were more lagged and had a longer warning time.

**Supplementary Information:**

The online version contains supplementary material available at 10.1186/s12889-022-14407-y.

## Background

Infectious diseases currently represent a major threat to human health. According to the National Health Commission of the People’s Republic of China, in 2019, the reported incidence of statutory infectious diseases was 733.57 per 100,000 and the reported mortality rate was 1.81 per 100,000 [1]. Infectious diseases are extremely diverse, with different routes of infection and complex influencing factors [2–4]. Humans exposed to the natural environment are therefore always exposed to infectious agents in the environment and within the human body, and the prognosis of infected populations varies depending on factors such as personal characteristics and the medical environment [5–7]. At the same time, as vaccines for some infectious diseases are still being developed, interventions in the transmission pathways of diseases are the main means of preventing the onset of infectious diseases [8–11]. Therefore, it is important to know when to start preventive measures against any given diseases, in order to prevent outbreaks of infectious diseases in time and to promote the optimal use of public health resources.

The main methods that can be used to model and predict the prevalence of infectious diseases are statistical models, individual random models, logistic differential equation (LDE) models and transmissibility dynamics models [12–19]. As the LDE models have an S-shaped curve, it can be used to describe the trend of “fast-slow-fast” in the cumulative number of incidences in the population during the spread of infectious diseases, so it is possible to calculate the point at which the epidemic starts to accelerate, the point at which it reaches its peak, and the point at which it decreases. It is therefore particularly suitable for modelling the fluctuations in the epidemiological profile of acute infectious diseases with seasonal and cyclical epidemics at each outbreak during the course of the epidemic [20–22]. At the same time, the LDE model is easy to understand and simple to calculate, making it suitable for the health sector to provide early warning of high incidence and seasonal fluctuations of acute infectious diseases. The model can also provide timely early warning signals, which can effectively control disease outbreaks and avoid wastage of medical resources.

According to previous research [23, 24], the LDE model is mostly used to explore the fitting and early warning studies of certain infectious diseases, but the application of the model to early warning of infectious diseases requires a symmetrical distribution of disease data. Due to the timely intervention of preventive and control measures during infectious disease outbreaks, the epidemiological curves of infectious diseases in most cases do not strictly conform to a symmetrical distribution, thus leading to errors in the determination of warning times. To solve this problem, a shape parameter *λ* is added to the LDE model in this study to improve the accuracy of the model, and the adjusted model is referred to as the generalized logistic differential equation (GLDE) model [25–27]. At the same time, the GLDE model is applied for the first time to the main functions of fitting and early warning of the incidence of infectious diseases in Jilin Province, and the model is well validated. For the selection of diseases, the actual incidence data of acute infectious diseases with seasonal and cyclical characteristics among 22 infectious diseases with different routes of transmission in Jilin Province were selected for 15 years. The LDE and GLDE models were applied to fit the incidence curve of the same acute infectious disease and estimated its warning week respectively. The difference in warning times calculated by the two models was compared to determine the optimal model for acute infectious disease warning using the logistic differential equation, to find the annual warning timeline for the province, to fill the gap that no study has yet conducted for multiple diseases in one area at the same time, and to suggest priorities for the implementation of prevention and control measures for different infectious diseases at different times and seasons in similar areas in the future.

## Materials and methods

### Study design

**Fig. 1:**
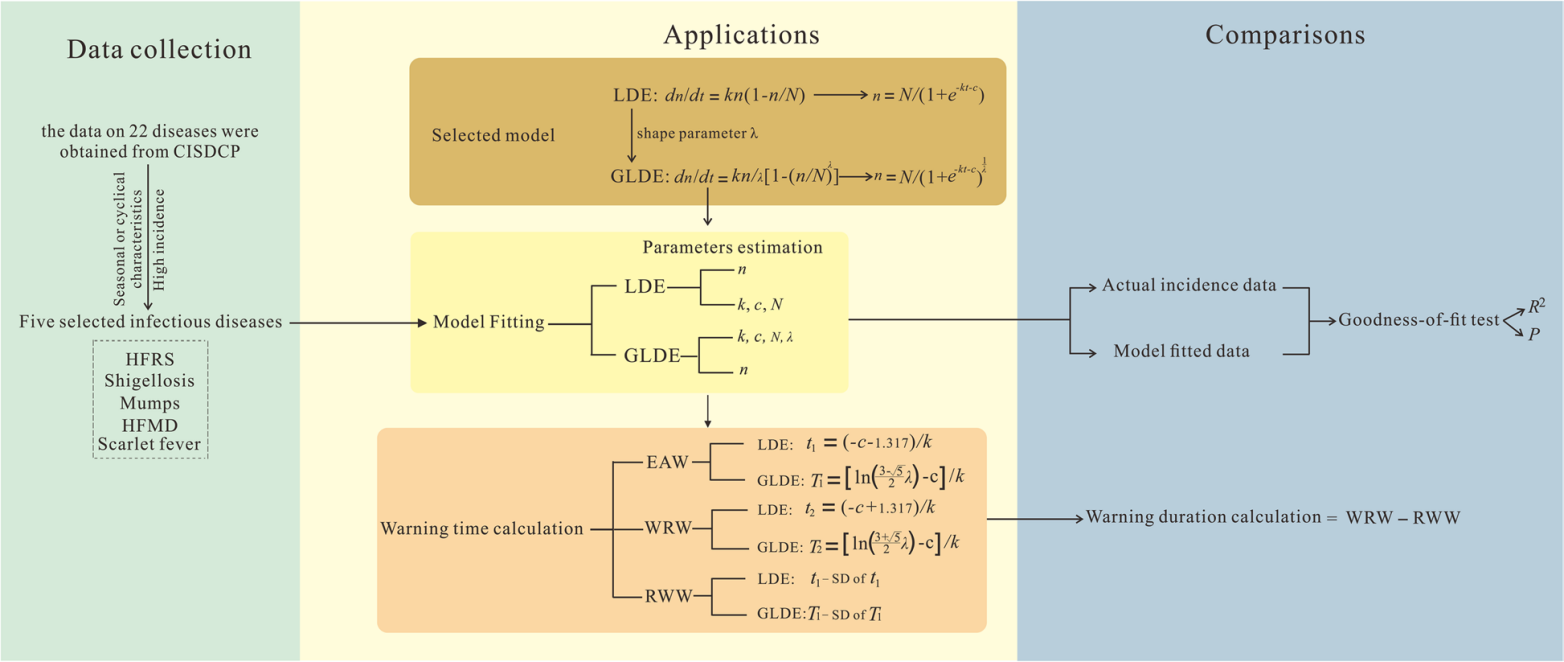
Research and design technology roadmap. (*n* is the cumulative number of infectious disease cases; *N* is the upper limit of cumulative infectious disease cases; *k* is the correlation coefficient; *c* is a constant; λ is a shape parameter; *SD* is the standard deviation; EAW is epidemic acceleration week; RWW is recommended warning week; WRW is warning removed week)

This study was conducted in accordance with the route of determining and constructing the LDE and GLDE models for the fitting and comparison of the effects of infectious diseases, and the estimation of warning times and comparison of differences between the two models. The GLDE is constructed by first introducing the shape parameter *λ* into the LDE. As LDE models were suitable for early warning of seasonal or cyclical diseases, acute infectious diseases with seasonal or cyclical characteristics were selected according to the weekly data collected for the prevalence and incidence of the disease. For the selected diseases, the epidemic cycle was segmented and the actual number of incidences (in weeks) was fitted using the two models respectively, and the goodness-of-fit test was performed on the data from the LDE and GLDE models. The parameters obtained in the fit were used to estimate the epidemic acceleration weeks (EAW) and the recommended warning weeks (RWW), and to compare the differences in warning durations estimated by the two models. The research design methodology involved is shown in Fig. 1 below.

### Data collection and criteria for inclusion and exclusion

In this study, the data on diseases were obtained from the China Information System for Disease Control and Prevention (CISDCP). Data were collected for 22 infectious diseases in Jilin Province from January 1, 2005 to December 31, 2019, where the data information included the date of disease onset. Diagnosis of all diseases followed the diagnostic criteria for infectious diseases developed by the National Health Commission of the People’s Republic of China. Demographic data were obtained from the Jilin Provincial Statistical Yearbook, including the total population, birth rate and death rate of Jilin Province for each year. The LDE models for early warning of the onset of chronic infectious diseases did not have practical application, and the LDE models were suitable for the early warning of seasonal and periodic infectious diseases. Therefore, in this study, the 22 diseases collected were classified into acute infectious diseases (HFMD, Mumps, Shigellosis, Scarlet fever, HFRS, Influenza, Rubella, Measles, Hepatitis A, Acute hemorrhagic conjunctivitis, Pertussis, Meningococcal meningitis, Typhoid and paratyphoid, Malaria) and chronic infectious diseases (Tuberculosis, Hepatitis B, Hepatitis C, Syphilis, Brucellosis, Gonorrhea, Hepatitis E, AIDs) according to their onset progression rate [28–30], and the acute infectious diseases with seasonal or cyclical characteristics were selected to be included in the fitting and early warning of the LDE models.

### Model building

#### LDE model

As early as 1845, Verhust proposed the LDE model, which is an ordinary differential equation (ODE) based on Malthus’ quantification of total biological growth to characterize the self-growth of disease in a population [16, 31]. In recent years the model has been widely used in the analysis of epidemiological characteristics of infectious diseases and the study of early warning mechanisms of infectious diseases [32]. Its main feature is the fitting of data to determine the particular specific time of the development of infectious diseases, with the following equation:

1 $$\frac{dn}{dt}= kn\left(1-\frac{n}{N}\right)$$

Where dn/dt is the rate of change of the cumulative number of infectious disease cases *n* at time *t*, *k* is the correlation coefficient and *N* is the upper limit of cumulative infectious disease cases. The general solution of eq. (1) is as follows:

2 $$n=\frac{N}{1+{e}^{- kt-c}}$$

This equation includes three parameters *k*, *N* and *c*. The meanings of *k* and *N* are the same as in eq. (1) and directly determine the trend of the cumulative number of cases *n* with *t*. The *c* is a constant calculated by integration during the solution of eq. (1) and is important when solving for the three inflection points of the logistic curve. The first order derivative of eq. (2) is expressed in terms of time *t*. The eq. (3) is as follows:

3 $$\frac{dn}{dt}=\frac{Nk{e}^{- kt-c}}{1+{e}^{- kt-c}}$$

The equation expresses the curve of new cases over time. If we take the derivative of eq. (3), which is the second order derivative of eq. (2), we can obtain an equation for the curve of the rate of increase or decrease in the number of new cases. The rate of change in the number of new cases is zero at the peak of the epidemic, so let the second order derivative of eq. (2) be equal to zero and solving for the inflection point from increase to decrease of the number of new cases i.e., solving for the value of t at the peak of the epidemic, where $t=-\frac{c}{k}$. The second-order derivative of eq. (3), which is the third-order derivative of eq. (2), gives the equation for the “acceleration” curve of the increase and decrease in new cases, and if this “acceleration” is equal to 0, the “acceleration” of new cases can be obtained. If this “acceleration” is equal to 0, the inflection point of the change in the “acceleration” of new cases can be obtained, as shown in eq. (4):

4 $$t=\frac{-c\pm 1.317}{k}$$

These two inflection points divide the process of infectious disease epidemic development into a gradual increase, a rapid increase and a slow increase, and the horizontal coordinate of the first inflection point corresponding to the gradual increase to the rapid increase is ${t}_1=\frac{-c-1.317}{k}$ [20]. The horizontal coordinate corresponding to the second inflection point from the fast to the slow growth period is ${t}_2=\frac{-c+1.317}{k}$ [20].

#### GLDE model

The GLDE model is improved to introduce the shape parameter *λ* into the LDE model, thus improving the model warning accuracy with the following differential equation:

5 $$\frac{dn}{dt}=\frac{kn}{\lambda}\left[1-{\left(\frac{n}{N}\right)}^{\lambda}\right]$$

Where $\frac{dn}{dt}$ is also the rate of change of cumulative infectious disease cases *n* at time *t*, the significance of the *k* and *N* parameters is consistent with the significance of the parameters in the LDE model above. Then the general solution of eq. (5) is as follows:

6 $$n=\frac{N}{{\left(1+{e}^{- kt+c}\right)}^{\frac{1}{\lambda }}}$$

The equation includes four parameters, *k*, *N*, *c* and *λ*, where *k* and *N* have the same meaning as in eq. (5) and directly determine the trend of the cumulative number of cases *n* with *t*. *c* is a constant resulting from the integration of eq. (5), which is important when solving for the 3 inflection points of the generalized logistic curve. *λ* is the shape parameter that determines the location of the distribution of the generalized logistic curve. When λ is greater than 0 and less than 1, the distribution is skewed to the left. When λ is greater than 1, the distribution is skewed to the right, and when λ is equal to 1, it is symmetrical, that is, the general logistics distribution. Expressing the first order derivative of eq. (6) in terms of time *t*, the eq. (7) is as follows:

7 $$\frac{dn}{dt}=\frac{kn}{\lambda }{e}^{- kt-c}$$

This equation expresses the curve of new cases over time. If we take the derivative of eq. (7), which is the second order derivative of eq. (6), we can obtain an equation for the rate of increase or decrease in the number of new cases. The rate of change in the number of new cases is zero at the moment when the epidemic reaches its peak, so let the second order derivative of eq. (6) be equal to zero and finding the inflection point at which there is an increase to decrease of the number of new cases, that is, the value of *T* at the peak of the epidemic, by solving for $T=-\frac{c+\ln \lambda }{k}$. The second-order derivative of eq. (7), which is the third-order derivative of eq. (6), gives the equation for the “acceleration” curve of the increase and decrease in new cases, and if this “acceleration” is equal to 0, the “acceleration” of new cases can be obtained as the inflection point for the change in “acceleration” of new cases is

8 $$T=-\frac{c-\ln \left(\frac{3\pm \sqrt{5}}{2}\lambda \right)}{k}$$

These two inflection points divide the development process of infectious disease epidemic into progressive, rapid and slow phases. The horizontal coordinate of the first inflection point from progressive to rapid phase is: ${T}_1=-\frac{c-\ln \left(\frac{3-\sqrt{5}}{2}\lambda \right)}{k}$, and the horizontal coordinate of the second inflection point from rapid to slow phase is ${T}_2=-\frac{c-\ln \left(\frac{3+\sqrt{5}}{2}\lambda \right)}{k}.$

### Simulation method and statistical analyses

In this study, diseases were fitted in segments according to the epidemiological cycle of the disease, based on the fluctuation of the disease epidemic curve. This was done using Berkeley Madonna 8.3.18 (developed by Robert Macey and George Oster of the University of California at Berkeley. Copyright©1993–2001 Robert I. Macey & George F. Oster) for modelling and the system of equations was solved using Runge–Kutta method of order four to find the best-fit curve and parameters. SPSS 21.0 (IBM Corp, Armonk, USA) was used to determine the goodness of fit of the model fit curve. The index for determining the goodness of fit was the root mean square (RMS) of the simulated and actual data [33, 34], and the larger the *R*^*2*^, the better the fit between the actual and simulated data and the test was *P* = 0.005.

### Establishing the timing of the warning

Equation (4) and Eq. (8) were used to calculate the two inflection points at which the speed of disease changes from slow to fast and from fast to slow in each epidemic cycle, namely the EAW and the warning removed weeks (WRW). As it takes time to implement health decisions and interventions and to produce the corresponding prevention and control effects, leaving the epidemic to develop until the “epidemic acceleration time” would result in a lag. Therefore, the mean and standard deviation (s) of the EAW for each epidemic cycle of the diseases were calculated. It is possible to consider an early warning time of 1–2 standard deviations ahead of the epidemic acceleration time, namely the RWW.

## Results

**Fig. 2:**
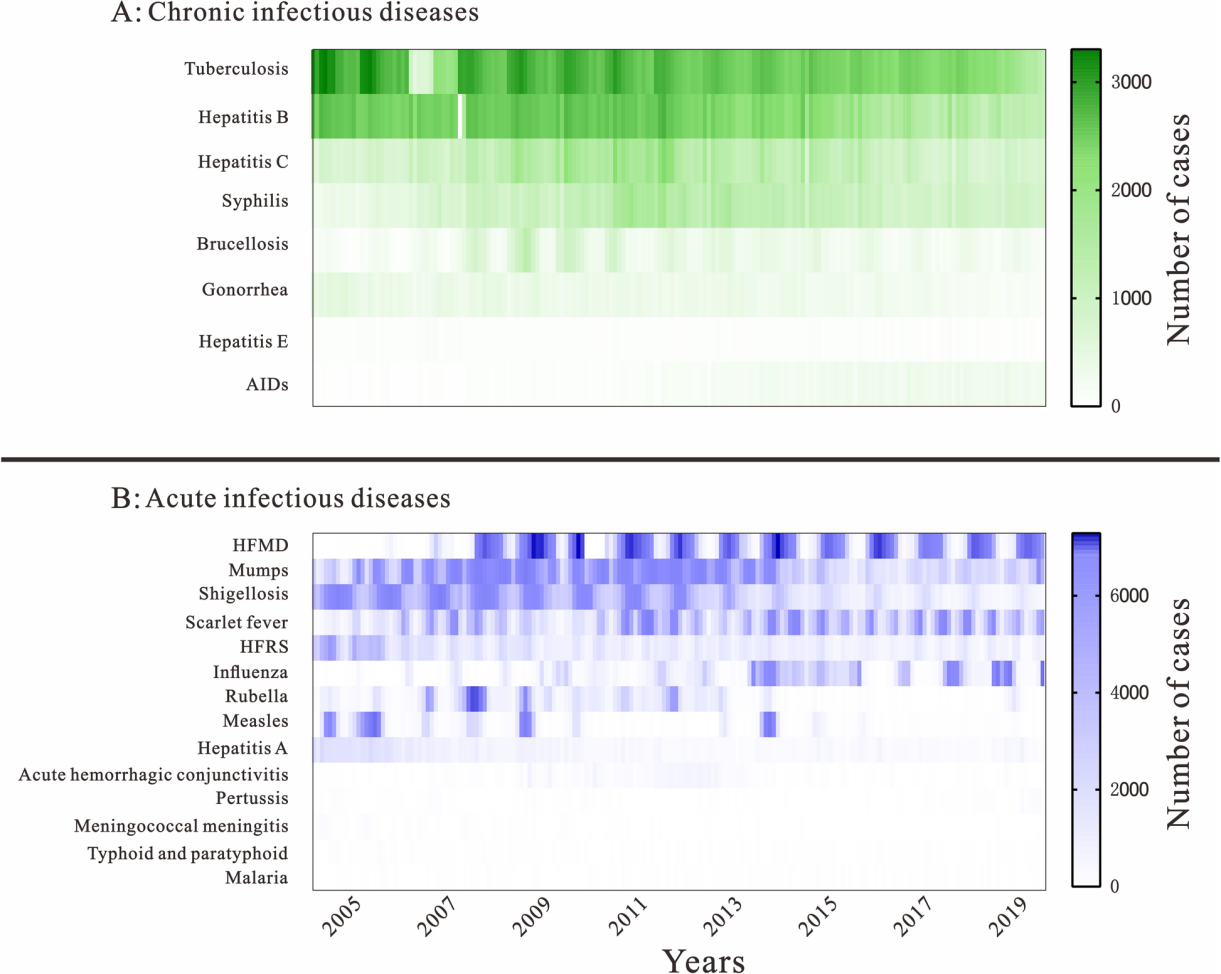
Overview of the incidence of 22 infectious diseases in Jilin Province, 2005–2019

Based on the incidence of 22 infectious diseases in Jilin Province, the cumulative number of cases of all infectious diseases collected in Jilin Province from 2005 to 2019 was found to be 928,530 cases. The average annual incidence rates of tuberculosis, hepatitis B and hepatitis C were higher among the chronic infectious diseases, at 69.17/100,000, 51.31/100,000 and 22.34/100,000 respectively. Among the acute infectious diseases, Hand, foot and mouth disease (HFMD), mumps, shigellosis, scarlet fever, and Hemorrhagic fever with renal syndrome (HFRS) had higher average annual incidence rates and were seasonal, at 38.17/100,000, 14.01/100,000, 10.43/100,000, 8.33/100,000, and 3.39/100,000 respectively, where mumps had only one peak (summer) in 2015 and 2016 respectively, and scarlet fever had one peak (summer) in 2009. Both diseases had two peaks in the remaining years (summer and winter); HFRS had one peak in 2015 (summer), three peaks in 2019 (spring, summer, winter) and two peaks in the remaining years (summer and winter); HFMD and shigellosis had one peak in each year from 2005 to 2019(summer). Based on the above, these five diseases were therefore selected for the calculation of early warning weeks. The results are shown in Fig. 2.

### Model fitting results of HFRS, shigellosis, mumps, HFMD and scarlet fever in Jilin Province

**Fig. 3:**
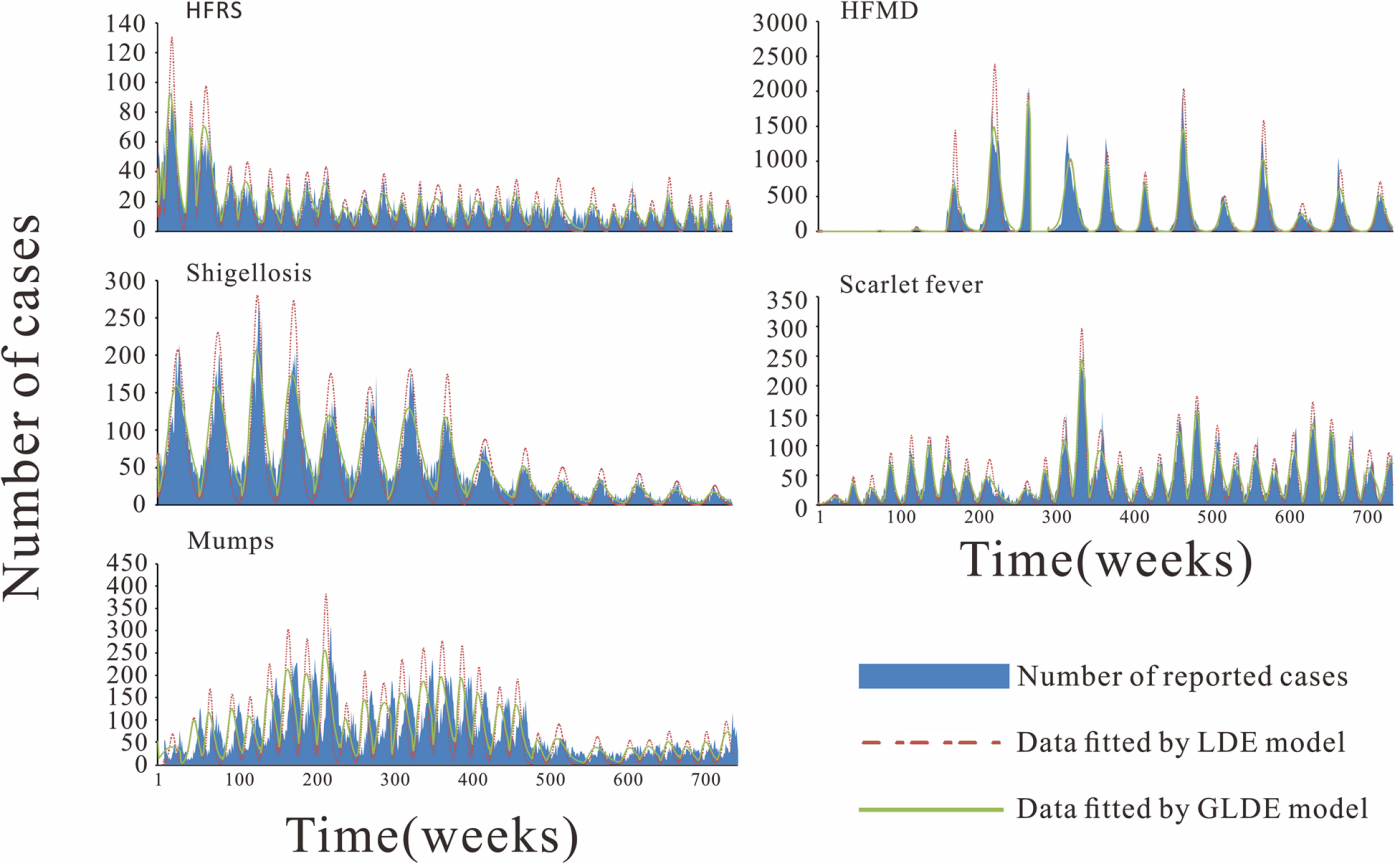
Fitted effectiveness of HFRS, shigellosis, mumps, HFMD and scarlet fever in Jilin Province, 2005–2019

The start of the epidemic cycle for HFRS, shigellosis, mumps, HFMD, and scarlet fever, i.e., the first week of 2005, was used as the starting time for LDE and GLDE models fitting. The results showed that the GLDE model fitted the epidemic data of these five diseases from 2005 to 2019 better than the LDE model, with values ranging from 0.80–0.94, and the differences were all statistically significant (*P* <  0. 005). The data simulated by the GLDE model were also closer to the actual number of reported cases. The results are shown in Fig. 3 and Table 1. The parameters *k*, *N* and *c* for each year during summer-autumn and winter-spring seasons for each disease fitted by the LDE model are shown in Additional file 1. The parameters *k*, *N*, *c*, *λ* for the GLDE model fitted for each disease for each year in the summer-autumn and winter-spring seasons are shown in Additional file 2.

**Table 1:** Goodness - of - fit test of HFRS, shigellosis, mumps, HFMD and scarlet fever in Jilin Province

Type of diseases	LDE		GLDE	
	*R*^2^	*P*	*R*^2^	*P*
HFRS	0.646	< 0.005	0.798	< 0.005
Shigellosis	0.856	< 0.005	0.877	< 0.005
Mumps	0.646	< 0.005	0.898	< 0.005
HFMD	0.846	< 0.005	0.937	< 0.005
Scarlet fever	0.869	< 0.005	0.879	< 0.005

### Establishment of recommended warning week and warning removed week

**Fig. 4:**
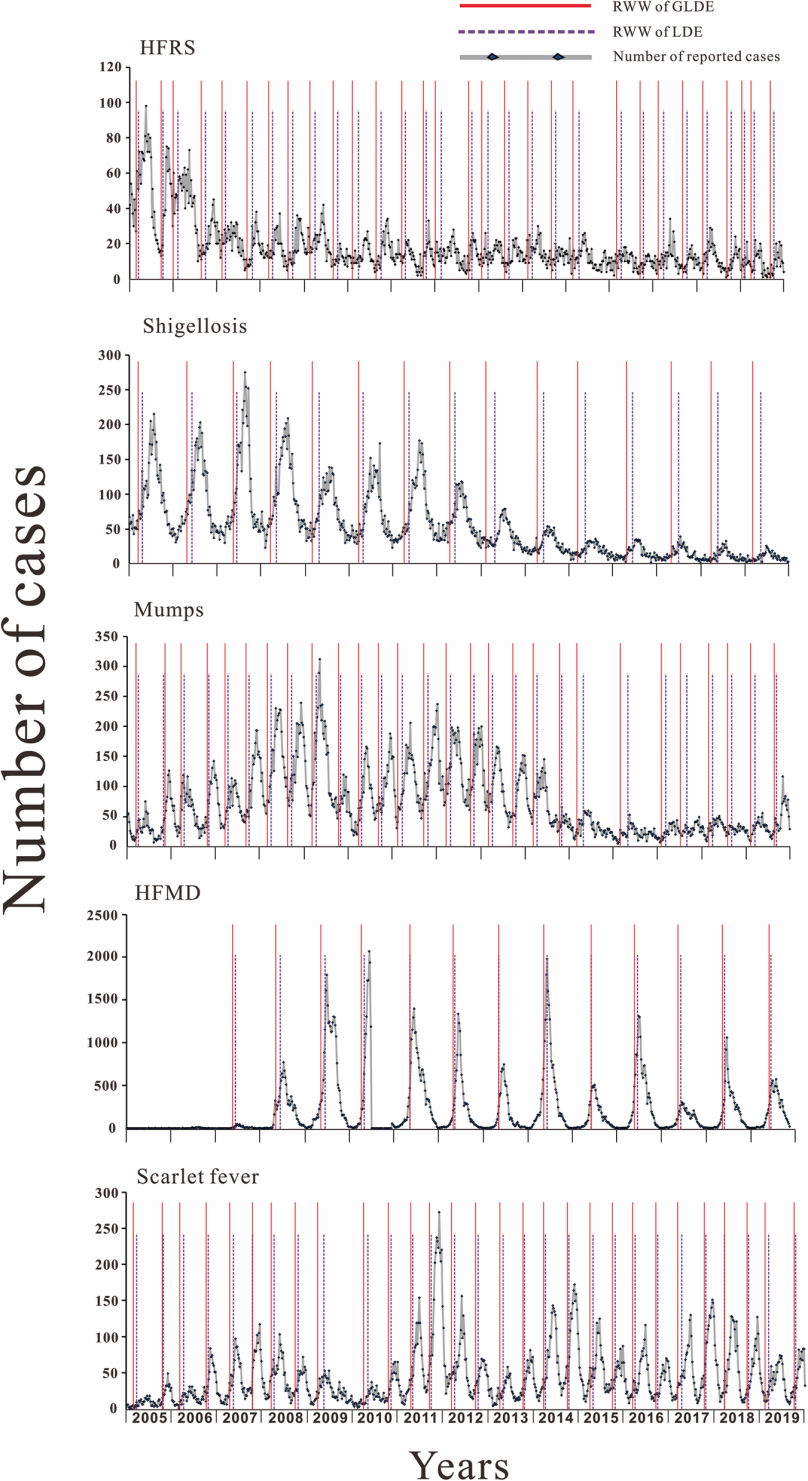
Early warning weeks for HFRS, shigellosis, mumps, HFMD and scarlet fever in Jilin Province in each year. (RWW is recommended warning week)

The parameters of the LDE model were brought into eq. (4), and the parameters of the GLDE model was brought into eq. (8) to calculate the EAW for each epidemic cycle of HFRS, shigellosis, mumps, HFMD, and scarlet fever in Jilin Province from 2005 to 2019, respectively. The mean EAW for HFRS in summer and autumn were approximately week 15 (range: week 12–18) and week 10 (range: week 7–14), with standard deviations of 3.31 and 3.69 weeks, respectively, while the mean values of the EAW in winter and spring were approximately week 43 (range: week 42–44) and week 40 (range: week 38–41), with standard deviations of 1. 28 and 1.45 weeks, respectively. The mean EAW for shigellosis in summer and autumn were approximately week 23 (range: week 21–25) and week 16 (range: week 13–19), with standard deviations of 2.37 and 3.03 weeks, respectively. The mean of the EAW for mumps in summer and autumn was about week 17 (range: week 15–19) and week 12 (range: week 11–14), with standard deviations of 1.87 and 1.72 weeks, respectively, while the mean of the EAW in winter and spring was about week 44 (range: week 41–46) and week 40 (range: week 37–44), with standard deviations of 2.72 and 3.70 weeks, respectively. The mean EAW for HFMD in summer and autumn were approximately week 27 (range: week 24–30) and week 25 (range: week 23–27), with standard deviations of 2.78 and 1.98 weeks, respectively. The mean of the EAW for scarlet fever in summer and autumn was about week 18 (range: week 16–19) and week 14 (range: week 12–16), with standard deviations of 1.49 and 1.84 weeks, respectively, while the mean of the EAW in winter and spring was about week 43 (range: week 42–45) and week 42 (range: week 40–43), with standard deviations of 1.15 and 1.34 weeks, respectively. According to previous studies [31, 35], the RWW for HFMD and mumps should be 2 weeks earlier than the EAW, as it is better to implement outbreak control measures earlier rather than later. It is therefore recommended that the RWW for HFRS are pushed forward by 3 and 4 weeks for both summer-autumn and winter-spring respectively, and that the RWW for shigellosis are pushed forward by 2 and 3 weeks for summer-autumn respectively, The RWW for scarlet fever are pushed forward by 2 weeks for both summer-autumn and winter-spring respectively. The results are shown in Fig. 4.

**Fig. 5:**
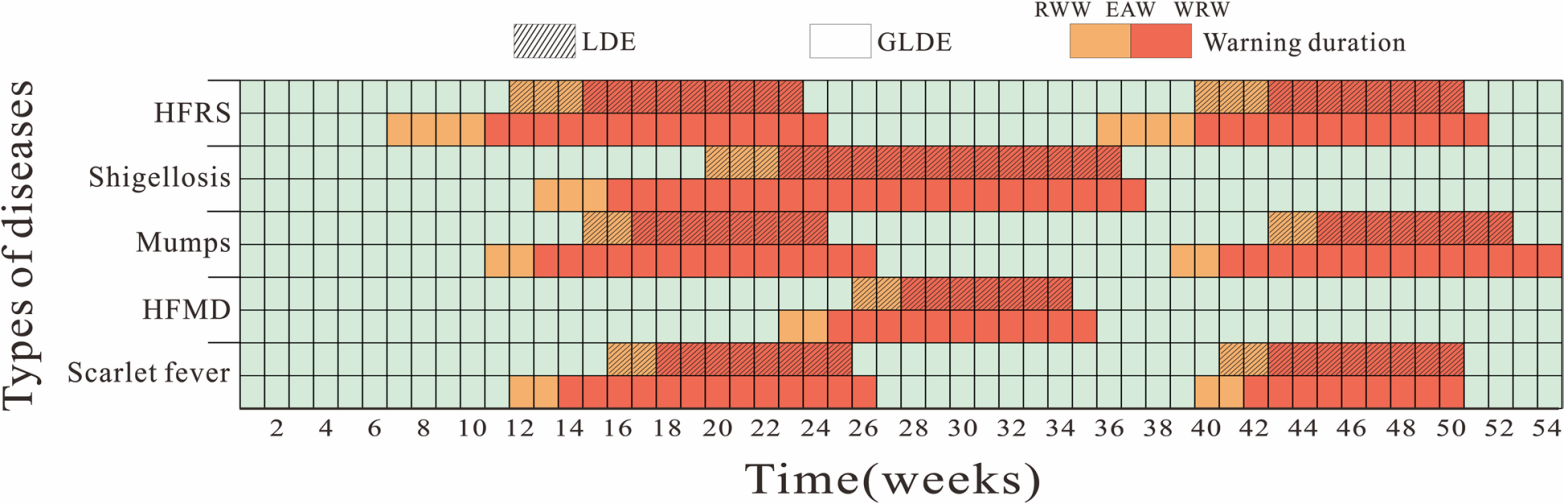
Duration of warning for HFRS, shigellosis, mumps, HFMD and scarlet fever in Jilin Province. (EAW is epidemic acceleration week; RWW is recommended warning week; WRW is warning removed week)

Based on the median number of EAW and WRW for each disease at each seasonal peak, as derived from LDE and GLDE models, the early warning timeline for high seasonal incidence in Jilin Province was drawn in chronological order. Overall, the start of warning for these five diseases was calculated using GLDE model to be earlier than that of LDE model and the duration of warning was longer than that of LDE model. According to the LDE model, the EAW and WRW for these five diseases show that Jilin Province should be under the warning status of the above five infectious diseases from week 12 to 36 and week 40 to 52 of the year, with two warning periods for HFRS, mumps and scarlet fever, and one warning period for shigellosis and HFMD. HFRS was first warned in the 12nd week of summer-autumn and lasted for 12 weeks to end the warning and in the 40th week of winter-spring and lasted for 11 weeks to end the warning; shigellosis is first warned in the 20st week of summer-autumn and lasted for 17 weeks; mumps is first warned in the 15th week of summer-autumn and 43nd week of winter-spring and lasted for 10 weeks; HFMD is first warned in the 26th week of summer-autumn and lasted for 9 weeks. Scarlet fever is first warned in summer-autumn week 16 and winter-spring week 41 and ends after 10 weeks. According to the GLDE model for these five diseases, it can be seen that from the 7th week of the year to the 2nd week of the following year, Jilin Province should be under the warning status of the above five infectious diseases. HFRS was first warned in the 7th week of summer-autumn and lasted for 18 weeks to end the warning and in the 36th week of winter-spring and lasted for 16 weeks to end the warning; shigellosis was first warned in the 13th week of summer-autumn and lasted for 25 weeks to end the warning; mumps was first warned in the 11th week of summer-autumn and in the 39th week of winter-spring and lasted for 16 weeks to end the warning; HFMD was first warned in the 23rd week of summer-autumn and lasted for 13 weeks to end the warning; and scarlet fever was first warned in summer-autumn week 12 and lasted 15 weeks and winter-spring week 40 and lasted 11 weeks. The results are shown in Fig. 5.

## Discussion

Currently, there are a number of models used for the fitting and early warning of infectious diseases, such as time series models, grey models and transmission dynamics models [18, 36–39]. The autoregressive moving average model (ARIMA) is one of the most common time series analysis and forecasting models, which can be combined with multiple models to analyze the stochasticity, smoothness and seasonality of time series data, and is suitable for short-term forecasting. Grey models are more commonly used in the fitting and prediction of infectious diseases, requiring less raw data, and can better predict the epidemiological trends of infectious diseases in the short term; transmission dynamics models build mathematical models that reflect the dynamics of infectious diseases based on their occurrence, transmission and development patterns within populations, and show the development process of diseases as well as reveal their epidemiological patterns through quantitative analysis and numerical simulation of the models. The models are used to show the development of diseases, reveal their epidemiological patterns and make short-term and long-term predictions. However, the above mathematical modelling methods are complicated to operate for grassroots disease control personnel, and the simulation results of the models require a solid theoretical foundation and extensive practical experience to make professional judgments, so they are less popular in the primary health care system. The logistic differential equation model is easy to understand, simple to calculate and can be used to estimate the point of inflection of the epidemic based on the results of the epidemic curve fitting, and adjust the intensity of preventive and control measures according to the warning time. Therefore, the early warning schedule for infectious diseases calculated in this study can be used as a theoretical reference for adjusting the timing of different prevention and control policies for different infectious diseases throughout the year in Jilin Province, and can then be extended to other regions with similar incidence of infectious diseases as Jilin Province. The model and methodology used in this study can also be applied to the calculation of early warning weeks for other diseases in other regions.

Due to the limitations of the LDE model, the data information required is more stringent, that is, the two segments of the waveform of its epidemic peak should be symmetrically distributed. However, in the disease process of many chronic infectious diseases there will not be obvious peaks and troughs, while even for diseases with more obvious seasonal fluctuations, when the infectious disease epidemic shows an upward trend, the intensity and effect of intervention measures taken by the health prevention and epidemiological departments will change with the progress of the epidemic. This in turn will lead to a change in the speed of the disease incidence trend, when the progress of the epidemic is not in line with the natural law of disease dissipation, and the waveform symmetry of the epidemic peak will change and the fit will become worse, resulting in the applicability of the LDE model being affected. This study addresses this problem by introducing a shape parameter λ into the LDE model and constructing a GLDE model to eliminate the effect of changes in the shape of the prevalence curve on the model fit and warning accuracy. To test the above hypotheses and to assess the applicability of the LDE and GLDE models, all statutory infectious disease epidemics in Jilin Province from 2005 to 2019 were selected for this study in order to compare the differences in the main applications of the two LDE models to infectious diseases. As chronic infectious diseases have a long incubation period, when epidemic fluctuations occur, this indicates that there has been a more widespread spread in the population, whereas the LDE models calculate the warning time based on the fluctuation curve of disease incidence, so for chronic infectious diseases warning and emergency prevention and control measures at the occurrence of a large number of cases does not have a better control effect on a large-scale spread that occurred a long time ago. Therefore, the need for early warning in an area is greatest for acute infectious diseases that are cyclical or seasonal in origin. In this study, data on the incidence of five selected seasonal and cyclical acute infectious diseases were counted on a weekly basis [40–45], and the results showed that for fitting the same disease, the GLDE model fitted better than LDE model. This indicates that the GLDE model can effectively adjust for the effects of fluctuations in infectious disease epidemiological trends that do not conform to symmetry, and therefore the GLDE model is more suitable for periodic or seasonal acute infectious disease incidence data.

The principle of the logistic differential equation model for early warning is mainly to calculate the inflection point of the change in speed when the epidemic fluctuates. By estimating the peak months of an epidemic based on past disease seasons, it is possible to avoid the spread of epidemics due to untimely warnings, as well as the waste of health resources due to a year-round state of prevention and control of the disease. In this study, the LDE and GLDE models were used to study the epidemiological characteristics of HFRS, shigellosis, mumps, HFMD and scarlet fever in Jilin Province during the period 2005–2019 and to determine the warning times for these five diseases in Jilin Province. Considering that the epidemic has already reached a high level by the time the EAW occurs, it means that there will be a lag in warning with this indicator. The RWW proposed in this study is a standard deviation before the epidemic changes from slow to fast early in the epidemic season, which is of great practical importance in preparing for the development and implementation of interventions. From the results obtain during an estimation of the early warning time, it can be seen that compared with the LDE model, the GLDE model has a longer warning duration for the same disease, with both the suggested RWW and EAW ahead, and the WRW lagging behind. This suggests that the GLDE model is more sensitive to the speed of change of epidemic curve fluctuations, and can calculate the warning signal in time when the epidemic starts to start slightly, thus more effectively avoiding the further spread of the epidemic. When the epidemic has completely subsided, the early warning elimination signal can be calculated to avoid the re-emergence of the epidemic due to the premature lifting of forced control measures. In disease early warning analysis, the GLDE model is therefore a more suitable early warning model under the regular prevention and control of infectious diseases. This means that early warning times are calculated for situations where the disease incidence curve waveform is asymmetrical, local health resources are more scarce and the severity of the disease is higher. Due to the shortcomings of the logistic differential equation model and the restrictions of the data, there are still some limitations in this study. The model is based on analysis of historical epidemiological data from Jilin Province and does not take into account the transmission dynamics of the disease. For instance, the epidemic cycles of HFRS, shigellosis, mumps, HFMD and scarlet fever are influenced by climatic conditions, and mumps and HFMD are also influenced by immunization levels, which were not considered in this study [46–50].

## Conclusion

For data on the incidence of acute infectious diseases that are seasonal or cyclical, the GLDE model is recommended for data fitting. LDE and GLDE models are both better at estimating and calculating warning schedules for these five highly prevalent acute infectious diseases in Jilin Province, but the GLDE model calculates a more accurate warning schedule. Overall, the recommended warning times estimated using the GLDE model were earlier than those calculated by the LDE model, the calculated warning removal times were more lagged, and the average warning duration was longer.

## Supplementary Information

**Additional file 1. **Fitting parameters of HFRS, shigellosis, mumps, HFMD and scarlet fever by the LDE model in Jilin Province. (*N* is the upper limit of cumulative infectious disease cases; *k* is the correlation coefficient; *c* is a constant).

**Additional file 2. **Fitting parameters of HFRS, shigellosis, mumps, HFMD and scarlet fever by the GLDE model in Jilin Province. (*N* is the upper limit of cumulative infectious disease cases; *k* is the correlation coefficient; *c* is a constant; λ is a shape parameter).

## Data Availability

The data that support the findings of this study are available from Jilin Provincial Centre for Disease Control and Prevention but restrictions apply to the availability of these data, which were used under license for the current study, and so are not publicly available. Data are however available from the authors upon reasonable request and with permission of Dr. Qinglong Zhao (jlcdczql@126.com).

## Abbreviations

LDE: Logistic differential equation
GLDE: Generalized logistic differential equation
RWW: Recommended warning week
EAW: Epidemic acceleration week
WRW: Warning removed week
HFRS: Hemorrhagic fever with renal syndrome
HFMD: Hand, foot and mouth disease
